# Mechanical resistance of the environment affects root hair growth and nucleus dynamics

**DOI:** 10.1038/s41598-024-64423-8

**Published:** 2024-06-14

**Authors:** David Pereira, Thomas Alline, Léa Cascaro, Emilie Lin, Atef Asnacios

**Affiliations:** Laboratoire Matière et Systèmes Complexes, Université Paris Cité, CNRS, UMR7057, 10 rue Alice Domon et Léonie Duquet, 75013 Paris, France

**Keywords:** *Arabidopsisthaliana*, Root hair, Nucleus dynamics, Mechanical resistance, Stiffness, Root-soil interactions, Biophysics, Cell growth, Nucleus, Plant sciences

## Abstract

Root hair (RH) cells are important for the growth and survival of seedlings. They favor plant–microbe interactions and nutrients uptake. When invading the soil, RH cells have to penetrate a dense medium exhibiting a variety of physical properties, such as mechanical resistance, that impact the growth and survival of plants. Here we investigate the effect of the mechanical resistance of the culture medium on RH-physical and phenotypical parameters such as length, time, and speed of growth. We also analyze the impact of the environment on nuclear dynamics. We show that the RH growth rate and the nucleus speed decrease similarly as mechanical resistance increases while the time of growth of RH cells is invariable. Moreover, during RH growth, the nucleus-to-tip distance was found to decrease when the stiffness of the environment was increased. Along this line, using Latrunculin B treatment in liquid growth media, we could internally slow down RH growth to reach speeds similar to those observed in stiff solid media while the nucleus-to-tip distance was only slightly affected, supporting thus the idea of a specific effect of mechanical resistance of the environment on nucleus dynamics.

## Introduction

Roots are important structures for plant anchorage and water uptake^1,2^. The external layer of the root in contact with the surrounding environment is called the epidermis. This layer is composed of atrichoblast and trichoblast cells, the latter differentiates in tubular structure called root hair cells (RHs). They are tip growing cells of 10 µm in diameter and can be few millimeters long^3^.

They are important for the plant soil interaction and nutrients uptake. Indeed, these tubular structures can increase drastically the surface of exchange of the root with the surrounding environment (up to twofold)^4^. In addition, they are important for root-substrate “cohesion” and soil erosion^5^, but also for root anchoring and penetration^6^. These cells have to evolve in heterogeneous environment presenting a variety of chemical and mechanical constraints. Depending on the soil strength (moistening, density, grain size and shape) the RH cells have to face a slightly or highly growth-resisting environment.

A key component of the root hair cell’s interaction with the surrounding environment is the cell wall (CW). It is a rigid structural component that acts like a barrier to maintain cell integrity by preventing cell from over-expansion/bursting under turgor pressure and protect it from damages due to mechanical stresses. RH cell growth is achieved through many coordinated processes: water uptake from outside to inside the cell, subsequent pressure increase, CW creeping at the RH tip (cell expansion) and new CW synthesis^7^. Thus, pressure drives RH growth and enables it to penetrate a mechanically resistant environment such as the soil. This process is called tip growth and has been also reported for many biological systems such as fungi and pollen tubes^8^.

Many studies focused on the growth of RH cells in liquid media under chemical or nutritive constraints^9,10^, but none were devoted to the potential effect of the mechanical resistance of the environment on the dynamics of RH growth. Thus, we decided to characterize the growth of root hair cells in mechanically resistant environments of different strength by growing RH in solid agar media of increasing concentrations (e.g. of increasing stiffnesses).

Beyond wall creep (expansion) at the RH tip, the nucleus plays an important role in RH growth in *Arabidopsis* *thaliana*^11,12^. On the one hand, it has been shown that the nucleus migrates inside the RH and is maintained at a fixed distance from the tip. On the other hand, blocking the movement of nucleus using optical tweezers was shown to reduce the growth rate of RH cells. Thus, nuclear movement and RH growth seem tightly linked. In fact, migration of the nucleus is also important for plant pathogen interaction, symbiosis, and could also be important for trichome development and for plant reproduction through male germ unit migration^13^. Thus, investigating the sensitivity of the nucleus movement and dynamics to external stimuli is of interest for many biological processes.

Indeed, it has been reported that the nucleus responds to light^14,15^ and mechanical stimuli. For instance, by using a needle, Qu and Sun have shown that the nucleus of epidermal leaf cell is sensitive to short time and repeated mechanical stimuli^16^. However, none has investigated the effect of mechanical cues on the dynamics of the nucleus in growing cells such as RH. Here we characterize the effect of RH growth in media of increasing stiffnesses on the position and movement of the nucleus, a first step in the understanding of mechanotransduction in the RH.

In the following, we first investigate the effect of the mechanical resistance of the surrounding environment on the growth of RH cells. We describe the repercussions of mechanical resistance on RH growth rate, length and time of growth. To do so, we used a microfluidic-like system (MLS) that allowed us to cultivate seedling for days, and to track RH cells over up-to 3 days^17^. We show that an increase in the mechanical resistance of the culture medium decreases the speed of growth and length of RH cells, but does not affect the time of growth.

Then, we describe the effect of the mechanical resistance of the culture medium on the nucleus dynamics, by looking at its speed of migration inside the RH, the fluctuations in position and speed, as well as the nucleus-to-tip distance. In particular, we show that the speed of the nucleus, as well as the distance between the nucleus and RH tip are reduced when the mechanical resistance of the environment is increased. Finally, using LatrunculinB treatment, we could test to what extend the relationship between the speed of growth and the nucleus-to-tip distance was specific to mechanical signaling. Indeed, 10 nM of LatB were enough to slow down RH growth -in liquid media- to speeds similar to those observed with the stiffest agar growth media. However, in contrast to the effect of external stiffness, LatB treatment led only to a slight modification of the nucleus-to-tip distance, suggesting that mechanical resistance of the environment has a specific impact on the nucleus dynamics of growing RH cells.

## Results

### The length and growth rate of root hair cells, but not the growth duration, decrease when the rigidity of the medium increases

To investigate the effect of the mechanical resistance of the environment on root hair cells’ growth, *Arabidopsis thaliana* seeds were grown in agar gels of three different concentrations (0.5% w/w, 1% w/w and 1.25% w/w). At higher agar concentrations, the number of RH cells decreased drastically (1.5%w/w) or RH were even unable to grow (2%w/w, Fig. S1a,b). Indeed, it was recently reported in agarose gels that raising the concentration increases the force necessary to penetrate the gels^18^.

In the following, we use the elastic moduli of the gels of different agar concentrations as a good proxy of their mechanical resistance to RH penetration. Thus, we measured the Young’s moduli of the gels varying agar concentration (Fig. S1). Young’s moduli were measured through a uniaxial compression experiment (see “Methods”).

**Figure 1:**
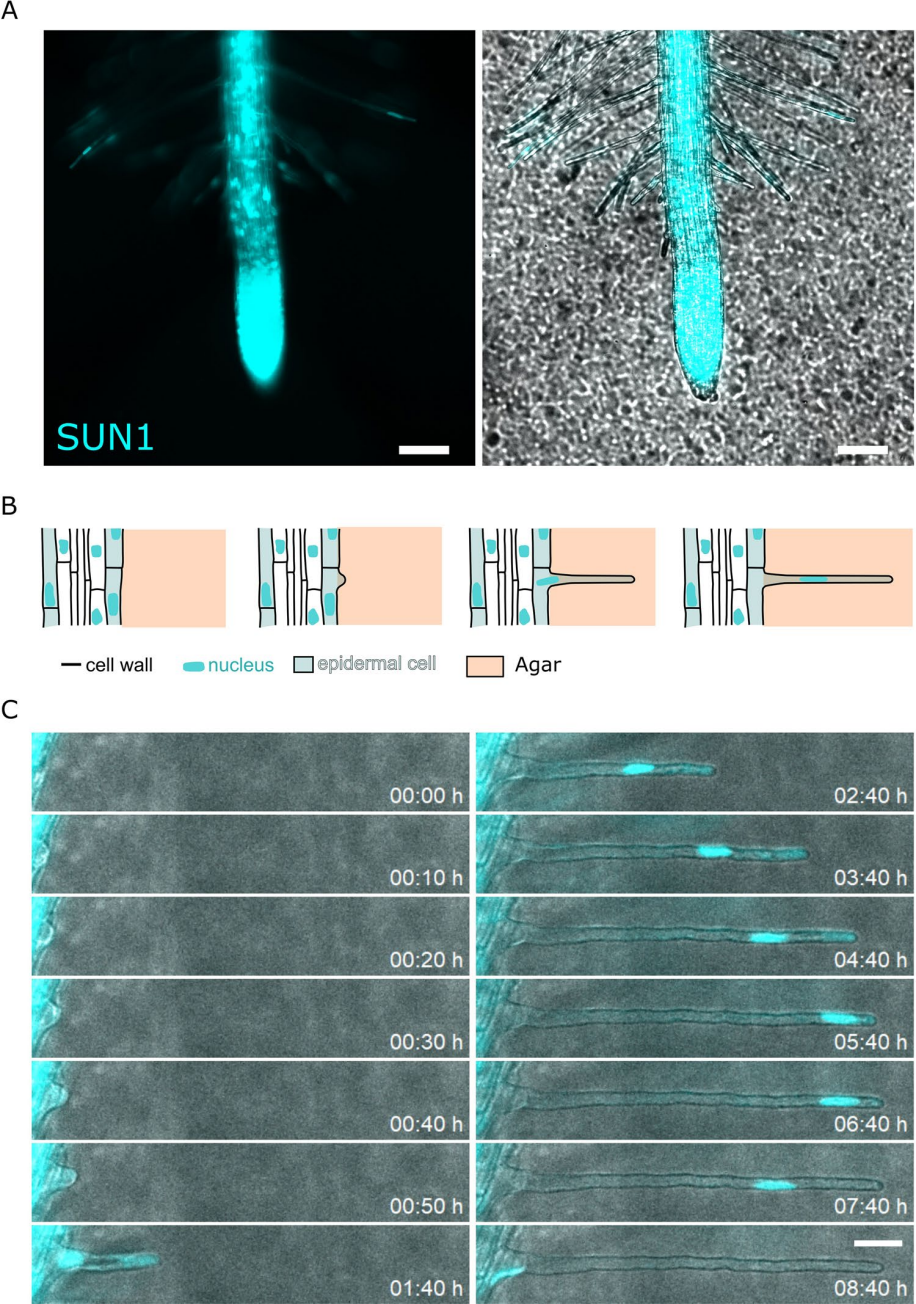
Root hair growth and nucleus imaging of Arabidopsis root and root hairs growing in 1/2 MS medium with 1% agar(w/w). (**A**) Left, fluorescence image of pSUN1:SUN1-GFP of a root and root hairs allowing to measure the nucleus position. Right, superposition of the bright field and of the fluorescence images (scale bar = 100 µm). (**B**) schemactic representation of a growing root hair. (**C**) Time-lapse of the growth of a root hair cell. Superposition of the bright field and of the fluorescence images (scale bar = 30 µm).

In order to monitor root hair cells growing for a long time period in agar gels, with a high temporal resolution, we used a microfluidic like system (MLS) that has been previously described^17^. After 5 to 7 days of growth, seedlings were placed under the microscope for imaging (Fig. 1A–C).

**Figure 2:**
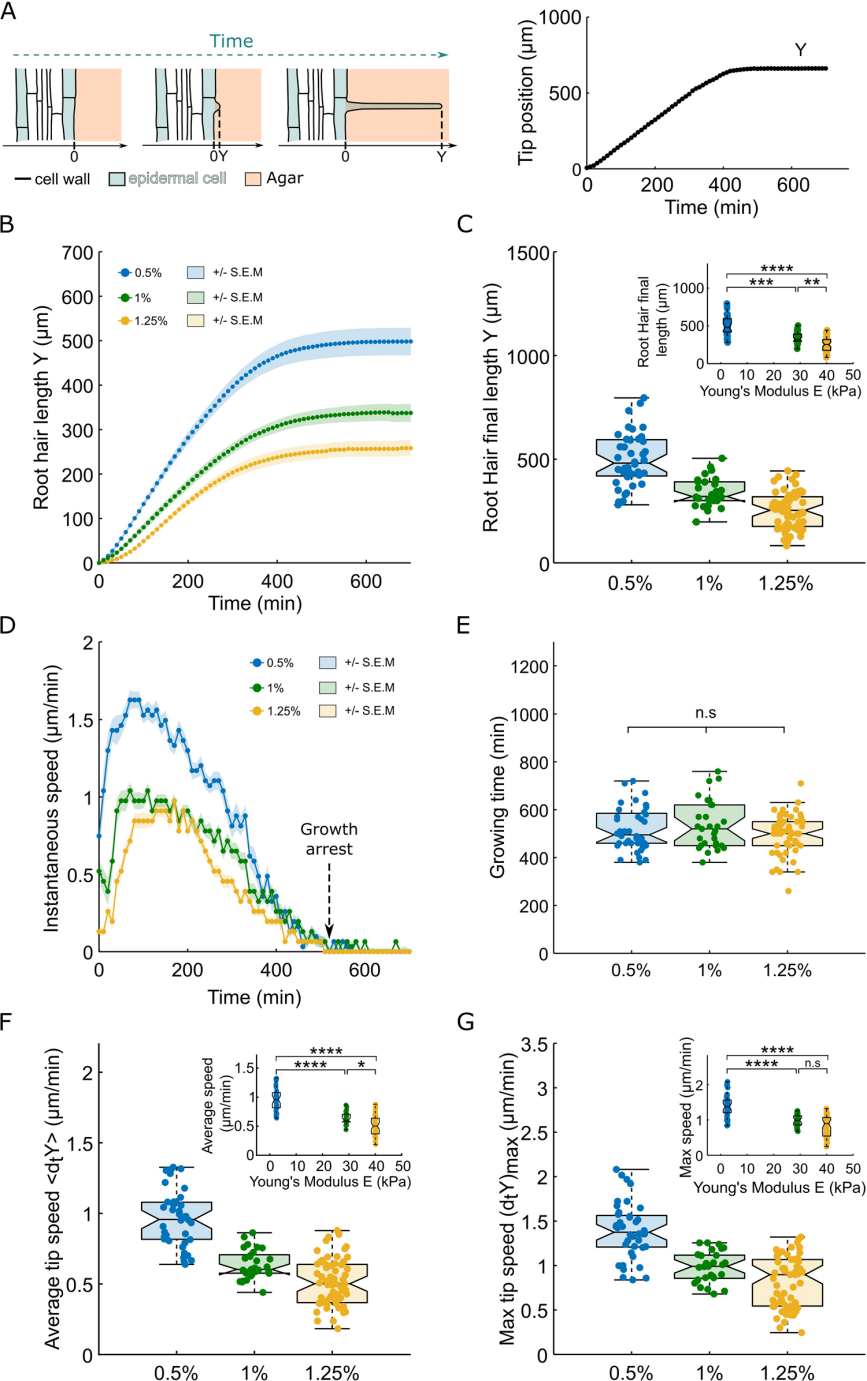
Kinetics of the root hair growth in agar of different concentrations. (**A**) Left, schematic representing the growth of a root hair at three different times. Y denotes the tip position. Right, representative example of the temporal growth curve of a root hair. (**B**–**D**) Growth features of root hair growing 1/2 MS medium with different agar concentrations, 0.5% agar (n = 40), 1% agar (n = 29) and 1.25% agar (n = 61). (**B**) Average root hair growth curves + /– S.E.M. (**C**) Boxplots showing the distributions of the final root hair length for the 3 agar concentrations, the inset is showing the same distributions as functions of the measured agar gel’s Young’s modulus. (**D**) Median instantaneous growth rate curves + /– S.E.M. (**E**) Boxplots showing the distributions of the total root hair’s growth time for the three agar concentrations. (**F**) Boxplots showing the distributions of the average root hair tip speed during the total growth time for the three different agar concentrations. The insert displays the same distributions as functions of the measured agar gel’s Young’s modulus. (**G**) Boxplots showing the distributions of the maximum root hair tip speed for the three different agar concentrations. The insert displays the same distributions as functions of the measured agar gel’s Young’s modulus.

To reveal the effect of agar concentration on the growth rate, the growth duration, and the length of RH cells, the position of the RH tip was measured over time (Fig. 2A) by applying a custom-made analysis pipeline on a kymograph of the Bright Field image (see “Methods”).

To measure accurately the final length of living root hair cells it is important to follow them over time to avoid bias such as dead cells or growing cells (Fig. 2A). For each cell, we measured the length after tip growth arrest. Root hair cell length was reduced by twofold when agar concentration was increased from 0.5 to 1.25%. (498 + /–20 µm, 339 + /–12 and 253 + /–11 µm for 0.5%, 1% and 1.25% agar, with 40, 29 and 61 cells, respectively) (Fig. 2B,C). Interestingly, the median value of the RH length at 0.5% is around 500 µm, which is relatively close to the ~ 580 µm reported for RH cells growing in liquid media^19^. This suggests that growth of RH in an agar gel of low concentration (0.5%) is similar to growth in liquid media. In contrast to the RH length, the duration of the RH growth was independent of the agar concentration, with a typical value of ~ 500 min (518 + /–14 min, 535 + /–18 and 495 + /–10 µm for 0.5%, 1% and 1.25% agar, with 40, 29 and 61 cells, respectively—Fig. 2D,E).

Since the RH length is reduced when agar concentration is increased, but not the time of growth, one expects the average speed of growth to decrease with the increase in agar concentration. Indeed, the analysis of the growth rates showed that the average speed of growth was reduced by twofold (from 0.96 + /–0.03 to 0.51 + /–0.02 µm/min) for agar concentrations ranging from 0.5 to 1.25% (Fig. 2F).

A careful examination of the growth rates shows that the mechanical resistance of the environment impacts each phase of the growth, from the initiation of the RH to the regular tip growth phase. Indeed, the average speed of the RH tip during the initiation phase was drastically reduced from 1.06 + /–0.03 to 0.48 + /–0.02 µm/min between 0.5 and 1.25% agar gels (Fig. 2B–D, Figs. S3, S4). During the rapid growth phase, the maximal speed of the tip was reduced by 40% when agar concentration was varied from 0.5% (1.39 + /–0.05 µm/min) to 1.25% (0.81 + /–0.03 µm/min) (Fig. 2G). In sum, we show that the speed and length of RH cells, but not the time of growth, are reduced by the increase of the mechanical resistance of the surrounding environment. Moreover, this effect is present from the initiation phase until the end of RH growth.

Finally, in order to confirm that the effect of the agar concentration was indeed due the mechanical properties of the gels, and not due to a putative change in solutes concentrations, osmotic measurements were performed and showed no significant differences between gels of different agar concentrations (Supplementary Information and Fig. S2).

### Increasing the mechanical resistance of the culture medium impacts the nucleus positioning and movement in root hairs

**Figure 3:**
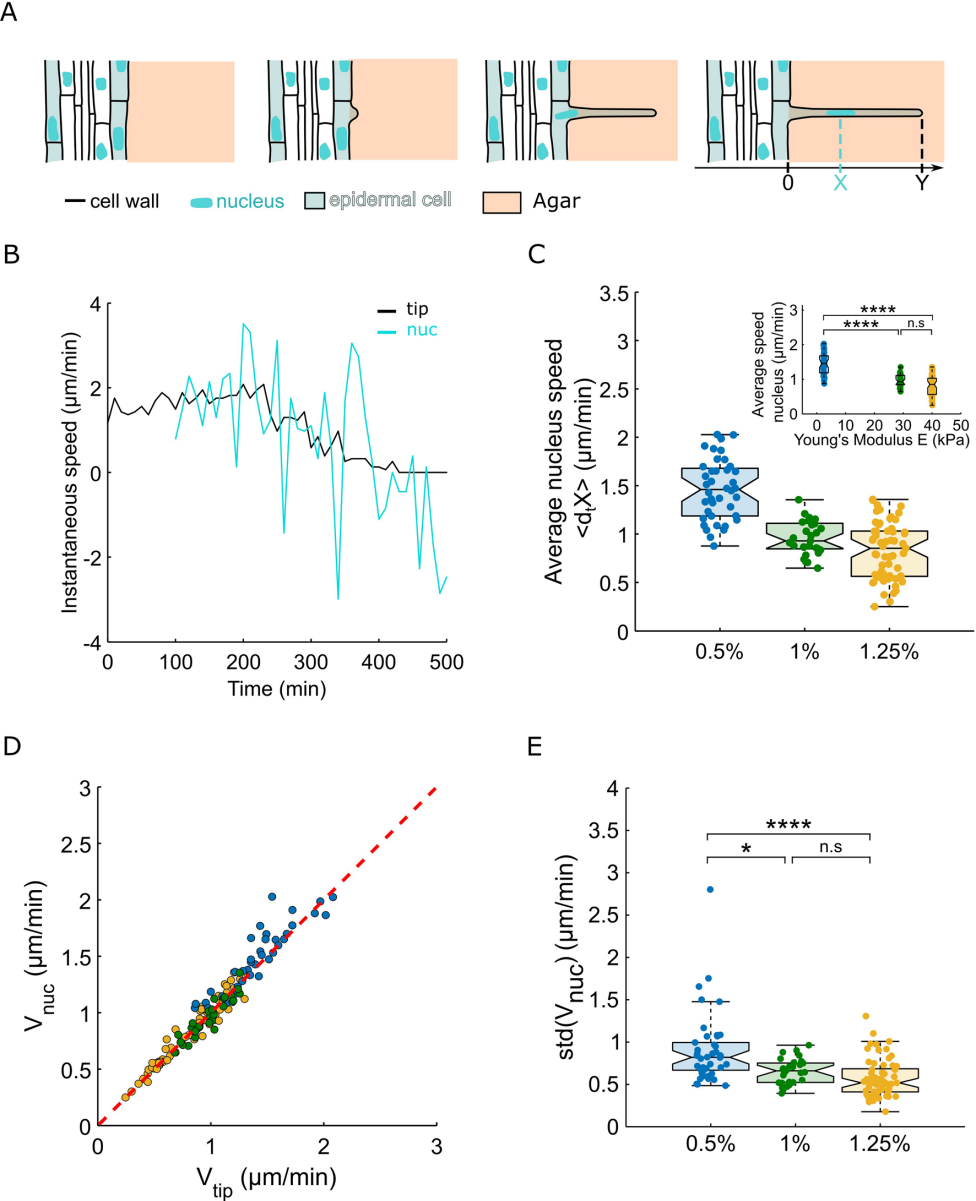
Kinetics of the nucleus of root hairs growing in agar of different concentrations. (**A**) Schematics showing the growth of a root hair and the positioning of its nucleus. Y denotes the tip position, and X the position of the nucleus along the root hair. The nucleus position is measured as soon as the nucleus enters the root hair. (**B**) Representative example of the growth speed of a root hair and the nucleus speed during growth. (**C**) Boxplots showing the distributions of the average nucleus speed during growth for root hairs growing in 1/2 MS medium with different agar concentrations, 0.5% agar (n = 40), 1% agar (n = 29) and 1.25% agar (n = 61), the inserts display the same distributions as functions of the measured agar gel’s Young modulus. (**D**) Boxplots showing the distributions of the standard deviation nucleus speed during growth for root hairs growing in 1/2 MS medium with different agar concentrations, 0.5% agar (n = 40), 1% agar (n = 29) and 1.25% agar (n = 61). (**E**) Graph showing the average nucleus speed as a function of the average tip speed, the red dotted line indicates where the two speeds are equal. The colors denote the agar concentrations (yellow, green and blue correspond to 1.25%, 1% and 0.5% agar concentrations, respectively).

Root hair growth and nucleus dynamics are connected^12^. Since we found that RH growth depended on the mechanical resistance of the surrounding environment, we decided to determine if nuclear dynamics could also be affected. Thanks to a cell line expressing pSUN1-GFP^20^, an inner nuclear membrane protein, we were able to image and track the nucleus of each cell during the whole RH growth, in gels of 0.5%, 1%, and 1.25% agar concentrations (Fig. 3A,B). The nucleus position over time was measured by extracting it from a kymograph of the GFP-signal (see “Methods”).

The nucleus speed (average speed during the RH linear growth phase) is drastically reduced (1.5-fold) when agar concentration is increased from 0.5 to 1.25% (corresponding to gels Young’s moduli ranging from 2.5 to 40 kPa) (Fig. 3C). In addition, the nucleus speed appeared to be nearly equal to that of the RH tip speed for the three agar concentrations tested (Fig. 3D).

It has been recently described that during the RH growth the direction of migration of the nucleus fluctuates^10,21^, it has a forward mean displacement but it can go backward. Very recently, Brueggeman et al. showed, by looking at the standard deviation of the speed of the nucleus, that the fluctuations of the speed increase when the microtubules are depolymerised. Looking at the standard deviation of the speed of the nucleus, we found that speed fluctuations decrease with the increase in agar concentration (Fig. 3E). However, by normalising the standard deviation by the average speed, we found that in the stiffer agar gels the nucleus speed fluctuations were less decreased than the speed of the nucleus itself (fluctuations-to-speed ratio increasing with the mechanical speed resistance of the environment, Fig. S5c). In sum, increasing the stiffness of the environment leads to “dampening” of the nucleus dynamics (e.g*.* reduced speed and fluctuations) even though speed and fluctuations are not decreased exactly in the same proportions (− 44 and − 37% respectively).

Interestingly, the effect of the rigidity appears at the very beginning of the RH growth, indeed, after the initiation step the nucleus entrance is delayed by the increase in rigidity. The nucleus enters in the tubular structure of the root hair after 80 min at low agar concentration, this time goes up to 120 min at high concentration (Fig. S6).

It has been previously described that the nucleus remains at a fixed distance from the RH tip during the fast growth phase^12^.

**Figure 4:**
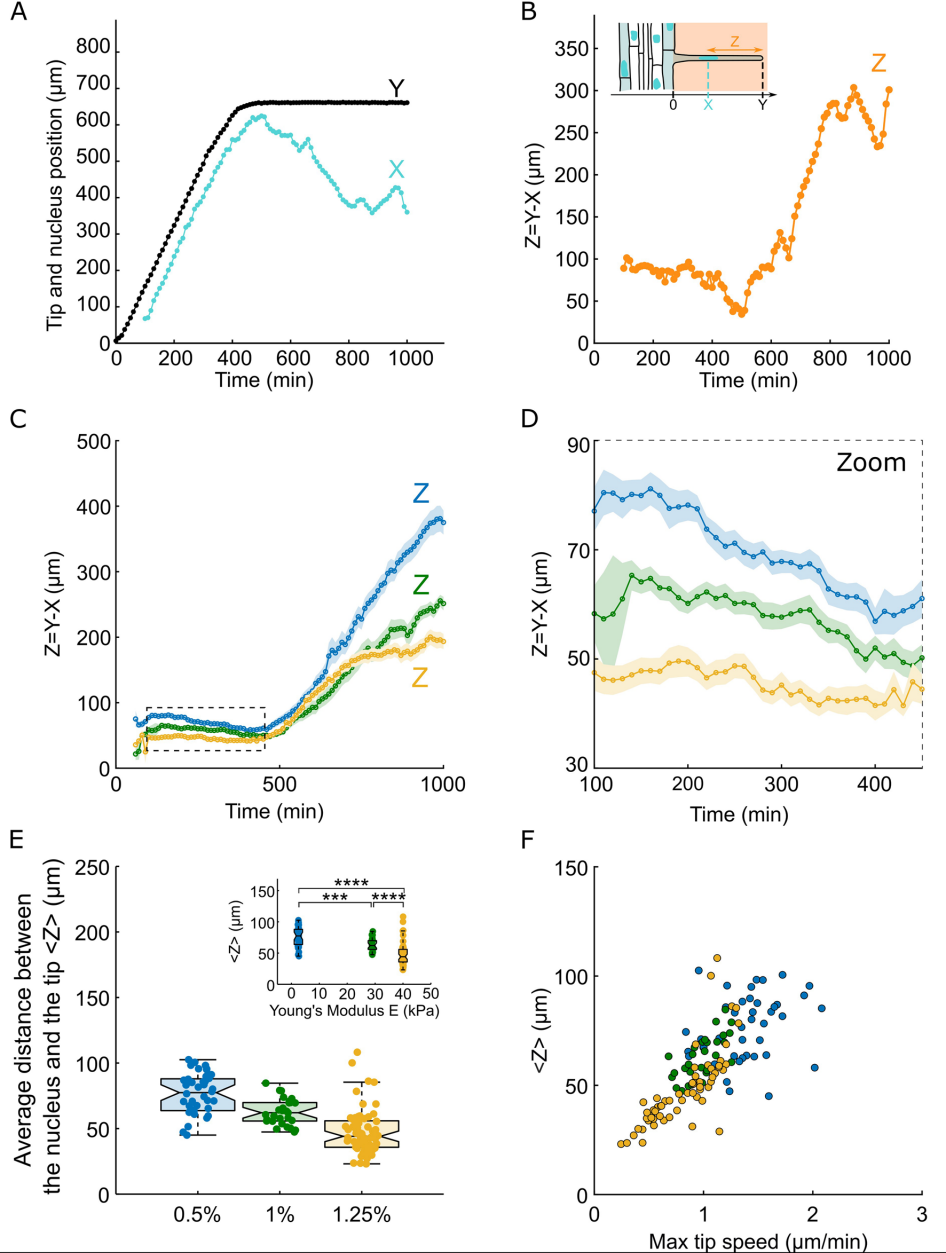
Nucleus positioning of root hairs growing in agar of different concentration. (**A**) Representative example of the growth of a root hair and the positioning of the nucleus during growth. (**B**) Distance between the tip and the nucleus in the example presented in (A). Z = Y–X denotes the tip-to-nucleus distance. (**C**,**D**) Average curves (+ /– S.E.M) of the nucleus-to-tip distance Z. (**E**) Boxplots showing the distributions of the average distance (calculated during the linear growth phase) between the nucleus and the tip for root hair growing in 1/2 MS medium with different agar concentrations, the insert displays the same distributions as functions of the measured agar gel’s Young modulus. (**F**) Graph representing the average distance between the tip and the nucleus as a function of the maximum tip speed. The colors denote the agar concentrations (yellow, green and blue correspond to 1.25%, 1% and 0.5% agar concentrations, respectively).

For every RH cell, the distance Z between the tip (Y) and the nucleus (X) was computed by doing the subtraction of X from Y: Z = Y-X (Figs. 4A,B and S7). The mean value of Z over the fast growth phase decreased from 76 + /–2 µm at 0.5% agar concentration (soft gel of 2.5 kPa) to 47 + /–2 µm at 1.25% (stiffest gel of 40 kPa), corresponding to a 43% decrease (Fig. 4C–E). Of note, the nucleus to tip distance Z shows some subtle trends. Indeed, unlike what has been previously reported^12^ Z is not strictly constant over time, but slightly decreasing (Figs. 4C,D and S8). Of note, while the current investigation is dedicated to the RH growth phase and its sensitivity to the mechanical resistance of the environment, it seems that the nucleus dynamics, after RH growth arrest, could also be sensitive to the stiffness of the environment (Fig. 4C).

In sum, the distance between the nucleus and the RH tip depends on the mechanical resistance of the culture medium: the stiffer is the environment the shorter is the distance between the nucleus and the tip (Fig. 4E). The nucleus to tip distance of 76 µm found for the 0.5% agar gels is similar to those reported previously for RH growing in liquid media^10,12^. This observation is in line with the fact that the RH growth speed in 0.5% agar gels was also similar to that reported for liquid media as mentioned in the previous section, indicating that 0.5% agar gels are soft enough to have similar impact on RH growth as liquid media.

In fact, along this line, it turns out that the rate of RH growth and the speed and positioning of the nucleus are similarly affected by the mechanical resistance of the culture medium, even quantitatively. Indeed, the distance Z between the nucleus and the tip appears to correlate well with the RH tip speed (Fig. 4F). This may suggest that the tip speed drives the nucleus-to-tip distance in root hair cells.

Since the RH tip growth rate and the mean speed of the nucleus are roughly the same, by calculating the ratio between the distance Z and the speed of the tip V_tip, one can have access to the lag time between the tip and the nucleus. Interestingly there is quite no effect of the mechanical resistance of the culture medium on this parameter (Fig. S9), the lag time is conserved.

Altogether, these results show that nuclear dynamics (speed, fluctuations, positioning) depends on the mechanical properties of the surrounding environment.

### Mechanical resistance to RH growth has significantly more impact on nuclear positioning than drug-driven slow-down of RH growth

**Figure 5:**
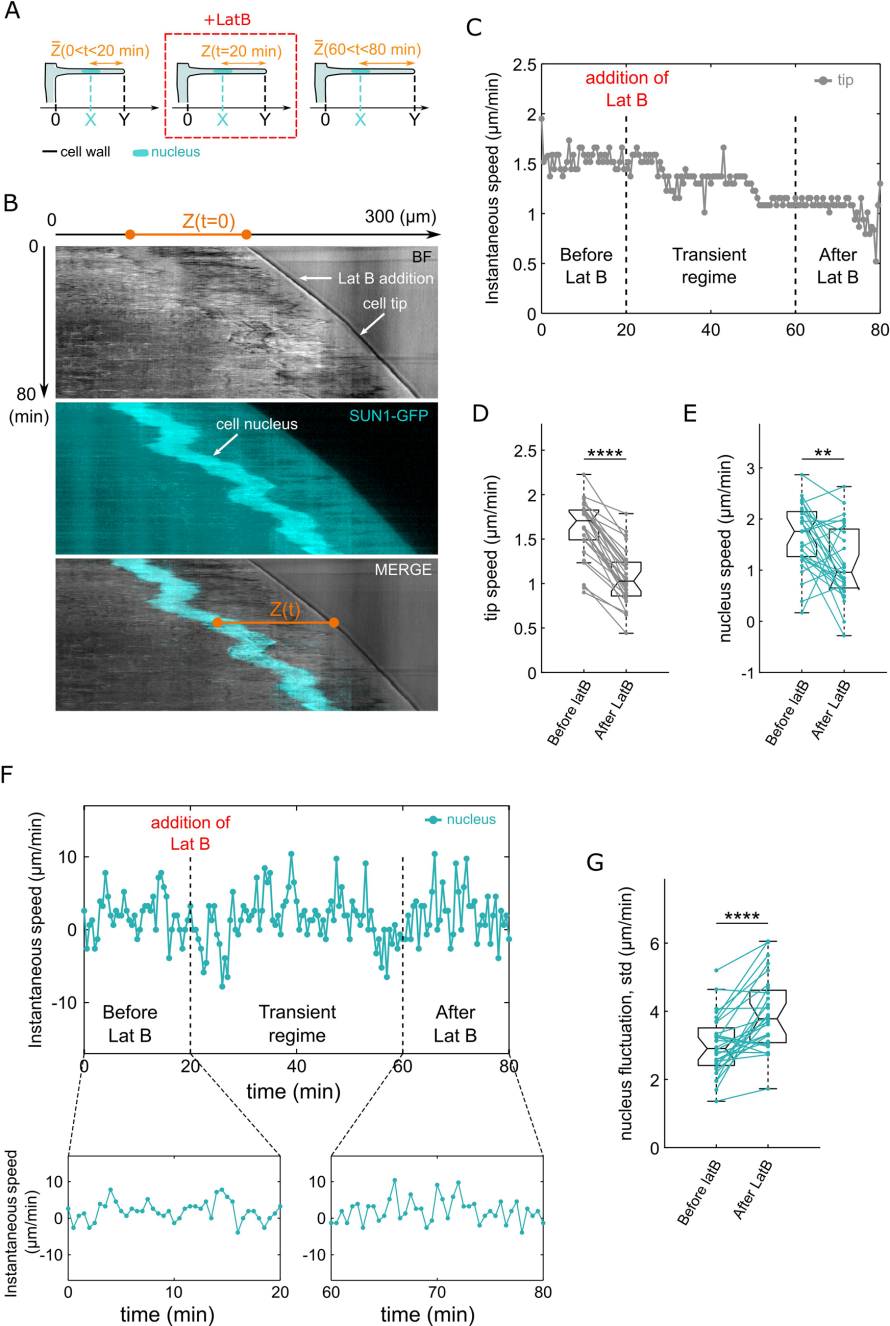
Root hair cells treated with Latruncunlin B have a reduced tip speed and show an increase in nucleus speed fluctuations. (**A**) Schematic representation of the Latrunculin B experiment. (**B**) Kymographs of a growing RH before and after treatment with 10 nM of LatB. (top) Bright field image showing the cell tip, (middle) image of SUN1-GFP signal showing the position of the nucleus over time, (bottom) superimposition of the two images. (**C**) Example of a quantification of the cell tip speed distance over time during LatB treatment. (**D**,**E**) Boxplots showing the distributions of the tip speed and nucleus-to-tip distance before and after LatB treatment (n = 28). (**F**) (Top) Example of the instantaneous speed of a nucleus during LatB treatment. (Bottom) Zoom on the durations during which the nucleus speed is quantified. (**G**) Boxplots showing the distributions of the standard deviation of the nucleus speed before and after LatB treatment (n = 29).

**Figure 6:**
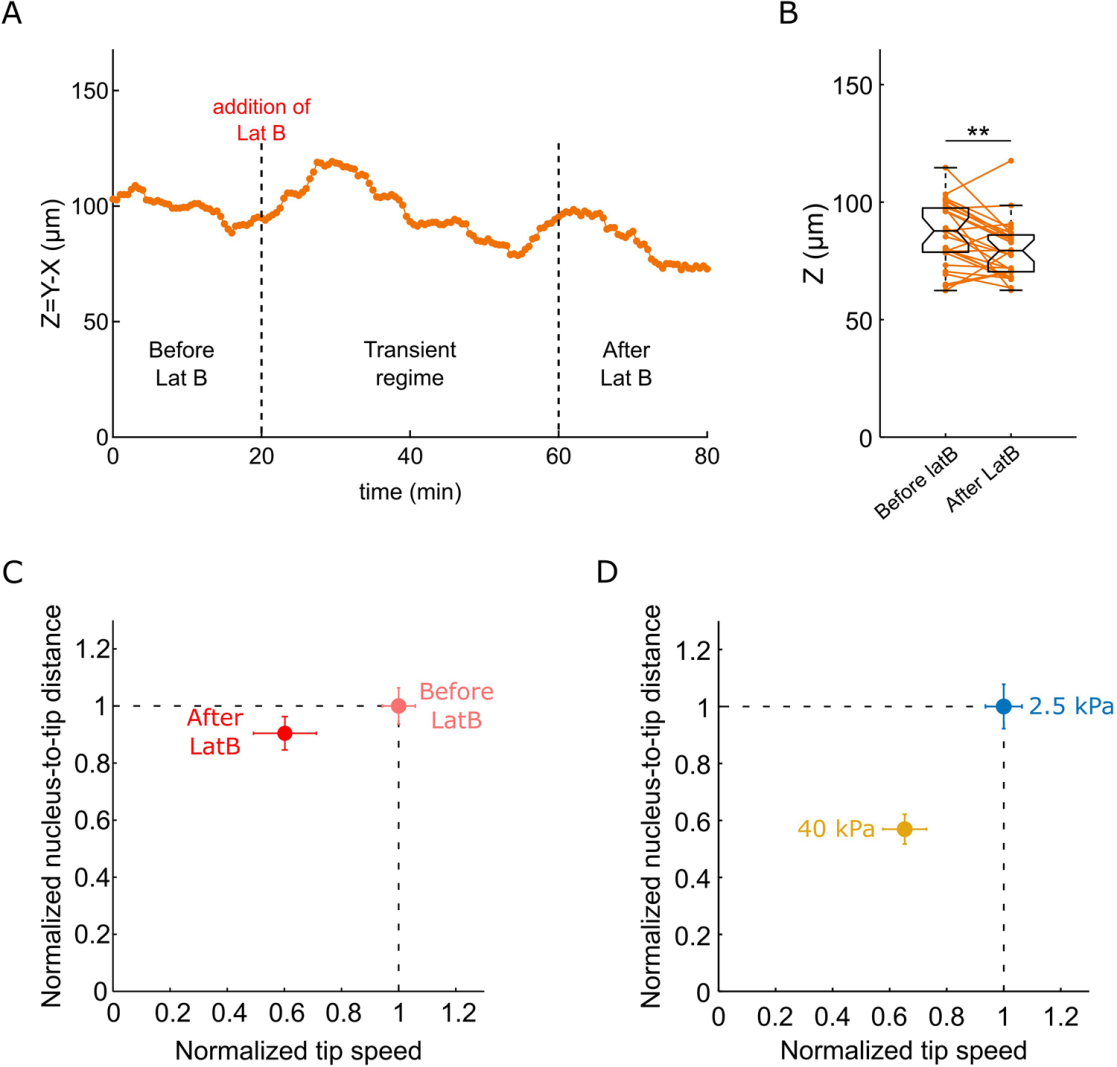
Root hair cells treated with Latruncunlin B show a slight decrease in nucleus-to-tip distance. (**A**) Example of the the nucleus-to-tip distance over time during LatB treatment. (**B**) Boxplots showing the distributions of the nucleus-to-tip distance before and after LatB treatment (n = 28). (**C**,**D**) Graphical comparison the effects of the environment stiffness and of LatB treatment on RH growth rate and tip-to nucleus distance. The normalized nucleus-to-tip distance is represented as a function of the normalized rate of growth, (**C**) before (reference) and after LatB treatment, and (**D**) in a soft (reference) and stiff environments. Data are normalized by the median, error bars represent the confidence interval (${q}_{2}\pm 1.57({q}_{3}-{q}_{1})/\sqrt{n}$, where *q*_2_ is the median, *q*_1_ and *q*_3_ are the 25th and 75th percentiles, respectively, and *n* is the number of cells).

To test the specifity of the effect of mechanical resistance on the nucleus dynamics (nucleus speed, fluctuations, poisition), we used Latrunculin B (LatB) which has been previously reported to affect root hair growth and nuclear dynamics through inhibition of actin polymerization^22,23^. We used a low concentration of Latrunculin B (10 nM) in order to avoid systematic growth arrest (Fig. S10). The RH speed, the nucleus speed and the nucleus-to-tip distance Z were measured before and after addition of LatB (10 nM) (Figs. 5A–F, 6A). The rate of growth was reduced by 35% (from 1.63 + /–0.06 to 1.06 + /–0.06 µm/min) after drug treatment (Fig. 5D). This decrease is similar to the one observed between the RHs grown in the softer and stiffer agar gels.

To quantify the effects of LatB on Nucleus dynamics, the speed of the nucleus and its standard deviation were measured before and after LatB addition. The nucleus speed decreased (from 1.65 + /–0.12 to 1.13 + /–1.13 µm/min) (Fig. 5E). Interestingly, the standard deviation of the nucleus speed increased (from 2.99 + /–0.17 to 3.91 + /–0.20 µm/min) (Fig. 5F–G). This increase shows an effect of LatB treatment on nucleus dynamics but it diverges from the effect of the mechanical resistance of the gels for which a decrease of growth speed comes with a decrease of nucleus speed fluctuations.

Finally, the LatB treatment led to an 8% decrease in nucleus to tip distance (from 87 + /–3 to 80 + /–2 µm) (Fig. 6A–C). This contrasts with the effect of the mechanical resistance of the environment. Indeed, as shown in the previous section, the stiffest agar medium leads to a similar decrease of the speed of RH growth, as well as of the nucleus-to-tip distance (from 1.39 + /–0.05 to 0.81 + /–0.03 µm/min, and from 76 + /–2 to 47 + /–2 µm), corresponding to 42 and 38% decrease, respectively (Figs. 6D and S11).

The control experiment with ethanol showed a smaller decrease in RH growth speed than with LatB (from 1.45 + /–0.07 to 1.17 + /–0.07 µm/min), and neither nucleus speed fluctuations nor positioning displayed significant changes (Fig. S12).

In sum, the mechanical resistance of the stiffest agar medium and a 10 nM LatB treatment lead to similar reductions of the RH growth rate, but mechanics has significantly more impact on nuclear positioning, and an opposite effect on the fluctuations of nuclear position. They also underline the importance of the mechanical properties of the environment in the regulation of nucleus dynamics.

## Discussion

Many previous studies demonstrated the importance of external cues, such as nutrients or water availability^24–26^ on the growth of root hair cells, but none looked at the effect of mechanical stimuli on root hair growth and the transduction of this signal to the nucleus. Our study represents the first step towards understanding the effects of mechanical stimuli on RH growth and their consequences on the nucleus dynamics.

The microfluidic-like system^17^ allowed us to control the mechanical properties of the surrounding environment of the RH cells. In addition, we were able to follow the growth of root hair cells from the initiation step to growth arrest. Notably, we observed that an increase in mechanical resistance of the culture medium led to a decreased length and speed of growth of root hair cells, while the time of growth was independent of the stiffness of the medium (Fig. 2), indicating that reduced RH lengths are due to reduced growth rates in mechanically resisting media. Interestingly, it has been recently reported in pollen tube that the speed of growth also depends on the stiffness of agarose medium. However, depending on the species, the speed of growth can either increase or decrease when the agarose concentration increases^18^.

The effect of medium mechanics on RH growth is quite different from the effect of chemical factors. For instance, Bates and lynch showed that a low phosphate availability in the medium increases the RH length and the time of growth^24^. Additionally, Datta et al. proposed that the transcription factor RSL4 determines the length of RH cells, the permanency of this protein is necessary to maintain the growth, when it disappears the RH cell stops growing^26^. Interestingly, the fact that in our experiments the time is constant over the three agar concentrations, make it tempting to speculate that RSL4 is not affected by mechanical cues. Thus, it would be interesting to look at presence of RSL4 in nuclei of RH cells under mechanically resistant environment.

A key component of the interaction of the root hair cell with the surrounding environment is the cell wall (CW). It is a rigid structural component that acts as a barrier to maintain cell integrity by preventing cell from bursting under osmotic constraints and protects it from damages due to mechanical stresses. The cell wall is mainly composed of cellulose, hemicellulose and pectin.

During cell growth, cell wall secretion and deposition is necessary to make possible cell expansion^7^. Considering that the growing time and the cell diameter are conserved but the not the length of RH cells, and if we hypothesize that the rate of cell wall deposition is the same under the different mechanical constraints, we could speculate that the cell wall thickness and/or structure could be different in highly resistant media as compared to slightly resistant media. Thus, it will be interesting to explore the thickness, the structure and the mechanical properties of RH cells that have grown under different medium resistances.

The ability of root hair to penetrate dense soil is critical to allow the plant to absorb more nutrients and to increase root penetration through an increased root anchorage^6^. Here, we report that an increase in external medium stiffness impede RH growth. In soils, root hairs do not grow in a homogenous medium as they can encounter stiff particles that can be much bigger than the RH diameter^27^. However, the reduction of growth described here can possibly lead to RH growth reorientation upon facing a local increase of rigidity. In other words, the RH growth reduction in stiff media could help the RH find the path of least resistance.

Thanks to a cell line expressing SUN1-GFP, we followed the dynamics of the nucleus from its entrance in the root hairs until the fully-grown step. Our experiments demonstrated the importance of the mechanical cues on the dynamics of the nucleus. As the mechanical resistance of the environment impaired drastically the RH growth, similarly, the nucleus speed was strongly reduced. Moreover, the fluctuations of the nucleus speed were modulated by the increase in mechanical resistance. Such nucleus sensitivity to mechanical cues was previously reported for non-growing cells, showing that a mechanical stimulus on trichome cell using a needle affects the nucleus positioning^16^. They showed a quick and transient response of the trichome cell nucleus upon a short time stimulation. Interestingly, in our experiments the nucleus response starts from the initiation step by a delayed entrance in the root hair, and is observed all along the RH growth through alteration of the nucleus migration speed, of its fluctuations, and of the nucleus-to-tip distance.

Ketelaar et al. have shown that the nucleus and the root hair tip are tightly linked, with a fixed nucleus-to-tip distance during RH growth. Here we show that the nucleus-to-tip-distance is dependent on mechanics, the distance being reduced for more mechanically resisting environments.

Many studies highlighted the role of actin in the link between the nucleus and the tip^11,12,21^. It has also been reported that the nucleus dynamics is an actin and myosin-XI dependent process^28^. Latrunculin is an actin-depolymerizing drug through actin monomer sequestration. Previous studies showed that a concentration of Latrunculin on the order of the µM arrests growth and inhibits nucleus movements^12^. However, a smaller concentration of LatrunculinB slows down RH growth in a dose dependent manner^22^. We thus applied a Latrunculin B treatment with a small concentration to perturb growth without completely disrupting the actin cytoskeleton. Our results show that a chemical perturbation through a 10 nM LatB treatment decreases RH growth. This treatment also affects nuclear dynamics and positioning by respectively increasing the nucleus speed fluctuations and decreasing the nucleus-to-tip distance.

Thus, LatB addition has a strong effect on root hair growth speed but a small one on nuclear positioning. This contrasts with the effect of the stiffness of the environment that have a strong impact on both root hair growth and nucleus dynamics (Table 1). These findings indicate that the RH growth rate and the nucleus dynamics are not necessarily linked, and suggest distinct mechanisms behind the effects of the mechanical resistance of the environment and that of chemical perturbations on nucleus positioning. Along this line, focusing on nuclear dynamics, it is noteworthy that increased stiffness of the environment and perturbation of the actin cytoskeleton lead both to a decrease of the nucleus speed, but induce opposite effects on the fluctuations of the nucleus speed, with stiffness leading to decreased fluctuations, while LatB treatment increases the fluctuations. Of note, Bruggemann et al. have shown that perturbation of the microtubule cytoskeleton through Oryzalin treatment also decreases the speed of RH growth and the speed of the nucleus, but increases the standard deviation of the nucleus speed, and has no effect on the nucleus-to-tip distance^23^ (Table 1). In sum, focusing on nuclear dynamics, it appears that depolymerisation of both microtubules (Brueggmann et al.^23^, Oryzalin treatment) and actin (current study, LatB treatment) decreases tip and nucleus speed (same effect as increasing stiffness), while both induce an increase of the fluctuations of the nucleus (opposite to stiffness effect). These observations suggest that nuclear dynamics involve a complex interplay between the MT and actin  cytoskeletons (csk). Altering one of these structures alters RH growth and nucleus displacement with increased fluctuations. In contrast, increased external stiffness seems to dampen nuclear dynamics as a whole, but not disrupting it.

**Table 1:** Effect of chemical and mechanical treatments on root hair growth parameters.

Experiment type	Tip speed	Nucleus speed	Nucleus-to-tip distance, Z	Nucleus fluctuations
Rigidity (0.5 to 1.25%)	− 42%	− 44%	− 38%	− 37%
Latrunculin B (10 nM)	− 35%	− 32%	− 8%	31%
Oryzalin (1 M); Brueggeman et al.^23^	− 36%	− 37%	9%	87%

Altogether, these results highlight the impact of the mechanical environment on nucleus dynamics, and transmission of mechanical cues from cell surface to the nucleus.

In this context, future studies should focus on the roles of the actin and the microtubule cytoskeletons (csk) in propagating and transducing mechanical stimuli from the cell surface to the nucleus. On the one hand, having shown that perturbation of the actin csk with LatB reduces the RH growth rate but not the positioning of the nucleus does not mean that the actin csk is not involved in the transmission of mechanical stimuli. This should be specifically tested in the future.

In conclusion, by using a custom-made microfluidic-like system, we were able to describe the effect of the mechanical resistance of the environment on the growth of root hair cells and the subsequent modification of nuclear dynamics and positioning, which could trigger mechanotransduction processes. Our results represent a first step in understanding the effect of mechanical cues on RH tip growth, implying in particular the regulation of the cell wall deposition, and/or the regulation of the cell’s internal pressure, paving the way for an integrated description and modelling of RH growth, and its adaptation to mechanical properties of the environment.

## Materials and methods

### Device preparation

The device used was prepared using a custom-made mould composed of few layers of cellulose acetate adhesive tape (3 M, Magic tape) with a single channel design of 250 µm height, 1 cm width and 2 cm length. PDMS 10/1 base/curing agent mixture (Sylgard 184, Dow Corning) was poured on the mould. The PDMS was cured at 65 °C for at least 4 h and peeled off the mold. The PDMS chips were then bounded to glass slide using a plasma cleaner (Harrick Plasma, PDC-002-CE).

The channel was filled with warm 1/2 MS medium (MS Murashige and Skoog) containing 0.5% sucrose (w/w) and 0.5%, 1% or 1.25% agar (Duchefa, plant agar, w/w) PH 5.7 and a 0.5 cm thick layer of the same warm 1/2 MS medium was deposited on the glass slide around the PDMS chip.

### Cell culture

*Arabidopsis thaliana*’s seeds expressing pSUN1:SUN1-GFP (an inner nuclear membrane protein) were used^20^. After sterilization, the seeds were stratified in an Eppendorf filled with 500µL of 1/2 MS medium (MS Murashige and Skoog) with 0.5% sucrose (w/w) PH 5.7 for 48 h at 5 °C. The seeds were subsequently transferred on the device and placed on the agar medium close to the entrance of the channel. The system was then placed in a Petri dish sealed with microporous film. Afterward, the Petri dish was placed with a 45° angle with respect to the horizontal in an incubator (Sanyo, versatile environmental test chamber MLR-351H). The plantlets were kept 5 to 7 days in the incubator with a 16 h light, 20.5 °C and 8 h dark, 17 °C cycle at 65% humidity in order to wait for the root to enter the main channel. Plants collection and culture were carried out following the relevant institutional, national, and international guidelines and legislation, with all the permissions and licenses.

### Microscopy/image acquisition

The device was placed in a microscope stage holder, few hundreds of microliter of ½ MS medium (MS Murashige and Skoog) with 0.5% sucrose (w/w) pH 5.7 were added on top of the system. Few milliliters of water were added around the system (not in contact with the agar medium) and a PVC chamber was placed above the system to limit evaporation and drying during the image acquisition. The images were recorded with a IX83 Olympus microscope equipped with a 20X0.45 NA objective. GFP fluorescence images were imaged with excitation light at 488 nm and the fluorescence emissions were recorded at 510 nm. For each plant, 42 fields of view were recorded every 10 min for 72 h using a motorized scanning stage (Marzhauser SCAN IM). For the Latrunculin B experiments, images were recorded every 30 s, using a 20X0.45 NA objective.

### Gels Young’s modulus measurement

Cuboid (l = 23 mm x l = 23 mm x L = 24 mm) of 1/2 MS medium (MS Murashige and Skoog) containing 0.5% sucrose (w/w) and 0.5%, 1% or 1.25% agar (Duchefa, plant agar, w/w) PH 5.7 were used to measure the gels’ young modulus. The gel samples were placed between 2 glass slides. A weight m was deposited on the top glass slide and the vertical displacement $\Delta {\varvec{L}}$ was measured using a 5X microscope objective. The strain was kept small (~ 1%). The young modulus E of the sample was then deduced from ${\varvec{E}}=\frac{{\varvec{\sigma}}}{{\varvec{\varepsilon}}}$ with ${\varvec{\sigma}}=\frac{{\varvec{m}}{\varvec{g}}}{{{\varvec{l}}}^{2}}$ the uniform vertical stress applied on the sample, and ${\varvec{\varepsilon}}=\frac{\Delta {\varvec{L}}}{{\varvec{L}}}$ the measured strain. For each agar concentration, 5 gels were measured.

### Image analysis

To measure the physical parameters of growing root hair cells, we developed a custom-made semi-automatized pipeline using ImageJ and Matlab softwares. For each RH cell two kymographs are extracted using ImageJ, one containing the position of the tip (bright field image) and the other one containing the position of the nucleus (image of the GFP channel). Then, using Matlab, the position of the nucleus is retrieved by finding, for every time point, the maximum intensity of the GFP signal. To extract the position of the tip of the RH cell on the bright field image, we draw a polyline on the kymograph close to the tip position then the algorithm searches for the minimum intensity near this line.

### Osmotic experiments

Solid media containing 1/2 MS were prepared with three different agar concentrations (0.5%, 1% and 1.25%). After the autoclave step and for each concentration, two tubes of 50 mL were poured with 5 mL of warm medium (each of them). After cooling down to room temperature, one tube was filled with 5 mL of ultrapure water and the other one with 5 mL of 1/2 MS medium. One empty tube was filled with 5 mL of ultrapure water and another one with 5 mL of 1/2 MS medium. In order to prevent evaporation, tubes were closed with a screw cap. After 24 h, the osmolality of the liquid phase of each tube was measured. Every experiment was repeated three times and three osmolality measurements were performed for each tube.

### Drug experiments

Root hair cells were grown in liquid medium (1/2 MS). 10 nM of Latrunculin B was added during RH growth. Approximately a quarter of RHs showed growth arrest upon Latrunculin B addition (Fig. S9). Only RHs that showed sustained growth for 60 min after LatB addition were analyzed. The average growth rate and nucleus-to-tip distance were measured over a period of 20 min, before addition of LatB (condition “before LatB”) and 40 min after LatB addition (condition “after LatB”).

### Statistical analysis

If not specified, the data are presented as mean values + /– s.e.m. Data presented are from at least 3 independent experiments. Statistical analysis was performed using either one-way ANOVA (Tukey–Kramer posthoc test) or KruskalWallis test (Dunn and Sidak posthoc test) depending on distribution properties. For normal distributions with equal variance or with at least 30 elements n (n ≥ 30 and equal variance), one-way ANOVA tests were performed, otherwise KruskalWallis tests were performed. Distribution normality and equality of the variance were evaluated using an Anderson–Darling and a Levene’s test respectively. For paired data, paired *t*-test were performed. The statistical analysis was carried out using Matlab. p-values are reported as non-significant for p > 0.05, or significant *p < 0.05, **p < 0.01, ***p < 0.001, ****p < 0.0001.

### Supplementary Information

Supplementary Information.

Supplementary Video 1.

## Data Availability

Data sets generated during the current study are available from the corresponding author on reasonable request.
